# Tumour shrinkage at 6 weeks predicts favorable clinical outcomes in a phase III study of gemcitabine and oxaliplatin with or without erlotinib for advanced biliary tract cancer

**DOI:** 10.1186/s12885-015-1552-y

**Published:** 2015-07-21

**Authors:** Seung Tae Kim, Kee-Taek Jang, Su Jin Lee, Hye-Lim Jang, Jeeyun Lee, Se Hoon Park, Young Suk Park, Ho Yeong Lim, Won Ki Kang, Joon Oh Park

**Affiliations:** 1 Division of Hematology/Oncology, Department of Medicine, Samsung Medical Center, Sungkyunkwan University School of Medicine, 81 Irwon-Ro Gangnam-gu, Seoul, 135-710 South Korea; 2 Department of Pathology, Samsung Medical Center, Sungkyunkwan University School of Medicine, Seoul, South Korea

**Keywords:** Early tumor shrinkage, Erlotinib, *KRAS*, Biliary tract cancer, GEMOX

## Abstract

**Background:**

The aim of this study was to determine whether early tumor shrinkage (ETS) at 6 weeks after treatment correlates with progression-free survival (PFS) and overall survival (OS) in advanced biliary tract cancer (BTC) patients receiving gemcitabine plus oxaliplatin (GEMOX), with or without erlotinib.

**Methods:**

This was a multicenter, open label, randomized, phase III trial of 103 BTC patients (ClinicalTrials.gov Identifier; NCT01149122, and Rigistration date; January, 7, 2010), comparing GEMOX with GEMOX plus erlotinib. Tumor shrinkage was expressed as a relative decrease compared to baseline and was dichotomized according to a previously reported cutoff value of 10 %.

**Results:**

Fifty-four patients (52.4 %) received GEMOX and 49 patients (47.6 %) received GEMOX plus erlotinib. The latter achieved a better overall response rate (RR) (40.8 % vs. 18.6 %, *p* = 0.02) and showed ETS more frequently (63.2 % vs. 40.7 %, *p* = 0.03). ETS was significantly correlated with the overall RR (correlation coefficient, 0.53; *p* < 0.01). The median PFS and OS did not differ according to erlotinib administration. However, the median PFS (7.3 vs. 2.1 months, *p* < 0.01) and OS (10.7 vs. 5.8 months, *p* < 0.01) were significantly longer amongst patients with ETS at 6 weeks after treatment, irrespective of erlotinib administration. In patients with wild-type *KRAS* who were treated with GEMOX plus erlotinib, ETS was a significant prognostic factor for PFS (*p* < 0.01).

**Conclusions:**

ETS might predict PFS and OS in BTC patients treated with GEMOX with or without erlotinib. Additionally, ETS may be an indication for adding erlotinib to chemotherapy for BTC patients wild-type *KRAS*. These findings need to be prospectively validated.

## Background

Biliary tract cancers (BTCs), including cholangiocarcinoma and gallbladder cancer, are relatively common in South Korea [1]. Because of the non-specific symptoms associated with these malignancies, more than 75 % of cases are unresectable as they are diagnosed an advanced disease stage. Moreover, even after complete resection, many patients experience disease recurrence. Patients with advanced or recurrent BTCs can be considered for palliative chemotherapy [2, 3]. Combination chemotherapy with gemcitabine and a platinum-based agent is regarded as a standard first-line chemotherapy regimen for advanced BTC, further to the results of previous randomized phase II and III trials (ABC01 and 02) [4, 5]. More recently, we conducted a phase III trial (NCT01149122) of gemcitabine and oxaliplatin (GEMOX) with or without erlotinib, a tyrosine kinase inhibitor that blocks epidermal growth factor receptor (EGFR) signaling. We found that the median progression-free survival (PFS) was 4.2 months in the GEMOX group and 5.8 months in the GEMOX plus erlotinib group [6]. These findings suggested that the addition of erlotinib to GEMOX might be considered as one of treatment options for BTC patients, although the difference in PFS between the groups was not significant.

Identifying patients who will derive the most benefit from treatment with a targeted agent is an important goal [7]. It is clear from the history of using anti-EGFR monoclonal antibodies (mAbs), including cetuximab, to treat cancer that not all patients benefit from these agents [8–10]. For example, colorectal cancer (CRC) patients with mutant *KRAS* tumors will not respond to or derive long-term benefit from treatment with anti-EGFR mAbs. However, no clinical characteristics or molecular biomarkers are currently available to identify subgroups of BTC patients who might survive longer if treated with a targeted agent. Previously, we evaluated the roles of *EGFR, KRAS,* and *PIK3CA* as biomarkers in patients with advanced BTC who received GEMOX with or without erlotinib, and found that *KRAS* status might be a predictive marker of response to erlotinib, but not of survival. Thus, further predictive markers of response and survival benefit after chemotherapy are urgently needed to facilitate the rational and effective use of drugs in cases of advanced BTC.

Rapid tumor shrinkage has been shown to be a surrogate marker of tumor EGFR dependency and consequently of cetuximab sensitivity, and several studies have reported that early tumor shrinkage (ETS) is associated with better long-term survival in metastatic CRC patients treated with anti-EGFR therapies [8, 11–13]. These findings also suggest that ETS may be a useful surrogate marker for making on-treatment decisions including continuation or discontinuation of therapy in daily practice. We hypothesized that adding erlotinib to chemotherapy could improve early tumor response in EGFR-positive BTC tumors. To investigate this hypothesis, we retrospectively analyzed clinical data from our previous randomized trial [6] and evaluated the predictive value of ETS for long-term outcomes in advanced BTC patients according to erlotinib treatment and tumor *KRAS* status.

## Methods

### Patients and samples

**Fig. 1:**
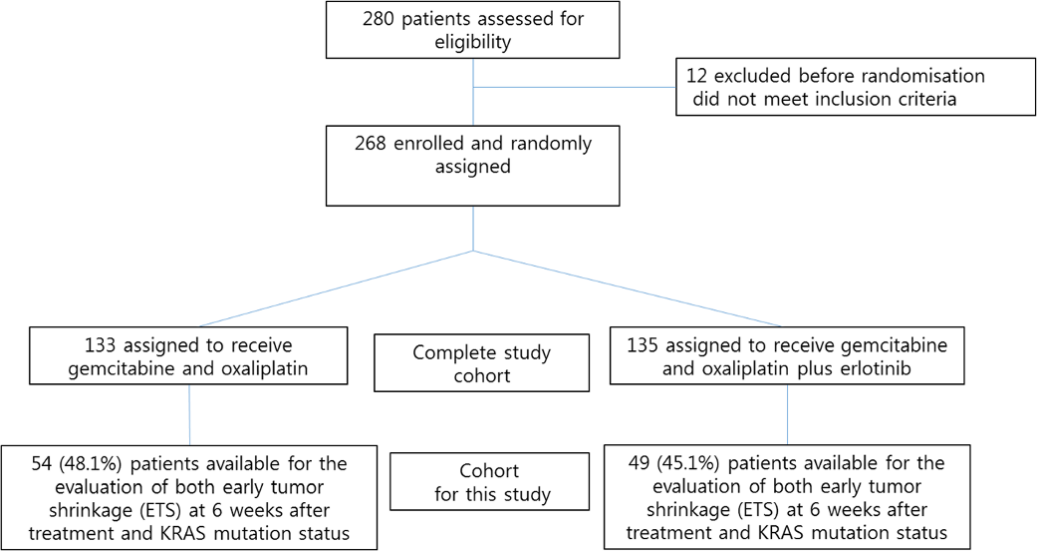
CONSORT flow diagram

The eligibility criteria and design of this study have been previously described [6]. Briefly, this was an open-label, randomized, phase III trial, in which 268 patients with advanced BTCs were randomly assigned to receive either erlotinib plus GEMOX (135 patients) or GEMOX alone (133 patients) as first-line treatment. All patients provided written informed consent according to institutional guidelines, and the study was approved by the Institutional Review Board. Tumor response was evaluated every 6 weeks using computed tomography (CT) and was assessed by the local investigators according to the Response Evaluation Criteria in Solid Tumors, version 1.0. A total of 103 patients were available for the evaluation of ETS 6 weeks after treatment as well as tumor *KRAS* mutation status (Fig. 1). Tumor shrinkage was expressed as a relative decrease compared to baseline and was categorized according to a previously reported cutoff value (10 %) [8, 12].

### Definition of ETS

Successive measurements of the target lesion were available for analysis. Changes in tumor size were expressed as a relative change of the sum of the longest diameter (LD) of the target lesions. ETS was calculated as the ratio of the sum of tumor LDs before treatment and 6 weeks after treatment. Patients who showed a reduction in tumor size of at least 10 % 6 weeks after treatment were considered to have achieved ETS.

### DNA Extraction and Mutation Analysis of *KRAS*

DNA was extracted from five 10-μm-thick formalin-fixed paraffin embedded sections containing a representative portion of each tumor block, using the QIAamp DNA Mini kit (Qiagen, Hilden, Germany). A pathologist (K.T.J) reviewed each slide and verified that more than 50 % of the tissue consisted of malignant cells.

Peptide nucleic acid (PNA)-locked nucleic acid polymerase chain reaction (PCR) clamping was carried out using the PNA-Clamp™ *KRAS* Detection kit (Panagene, Inc., Daejeon, Korea), as described previously. Briefly, the reaction mixture contained 10–25 ng template DNA, primer and PNA probe set, and SYBR Green PCR master mix in a total volume of 20 μl. All necessary reagents were included in the kit. Real-time PCR reactions of PNA-mediated PCR clamping were performed using a CFX 96 system (Bio-Rad, USA). PCR cycling conditions were a 5-min hold at 94 °C, followed by 40 cycles of 94 °C for 30 s, 70 °C for 20 s, 63 °C for 30 s, and 72 °C for 30 s. This method allowed 7 different mutations in exon 2 of the *KRAS* gene to be detected.

### Statistical analysis

Descriptive statistics were reported as proportions and median. The correlation between ETS and overall tumor response was evaluated using Spearman’s correlation. PFS was defined as the time from date of first study treatment to date of first documented disease progression or death. Overall survival (OS) was calculated from the first study treatment until death. PFS and OS were evaluated according to treatment and achievement of ETS, and differences were analyzed using the Kaplan-Meier method and stratified log-rank test. Additionally, for a subgroup of patients with wild-type *KRAS* tumors, Kaplan-Meier plots were constructed for PFS according to treatment and achievement of ETS and were compared using the log-rank test. Hazard ratios (HRs) were estimated using the Cox proportional hazards model. *P* < 0.05 was considered significant.

### Ethics statement

The Ethics Committee at Samsung Medical Center approved the study in accordance with the Declaration of Helsinki. All individuals gave written informed consent for participation in the study.

## Results

### Patient characteristics

This analysis included 103 patients who received GEMOX alone (*n* = 54) or GEMOX plus erlotinib (*n* = 49) as first-line treatment for advanced BTCs. Patient characteristics according to the treatment received are summarized in Table 1. The baseline patient characteristics did not differ significantly between the treatment groups (Table 1), with the exception of predominantly metastatic disease that occurred significantly more frequently in the GEMOX group than in the GEMOX plus erlotinib group (92.6 % vs. 63.3 %, *p* = 0.04).

**Table 1:** Characteristics of 103 advanced biliary tract cancer patients treated with gemcitabine and oxaliplatin (GEMOX) with or without erlotinib

	Study population	
	GEMOX (*n* = 54)	GEMOX plus erlotinib (*n* = 49)
Age, years		
Median	62 (45–75)	59 (39–75)
Sex		
Male	35 (64.8 %)	33 (67.3 %)
Female	19 (35.2 %)	16 (32.7 %)
Primary site		
Cholangiocarcinoma	38 (70.4 %)	38 (77.6 %)
Gallbladder (GB)	16 (29.6 %)	11 (22.4 %)
Differentiation		
Well/Moderate	33 (61.1 %)	26 (53.1 %)
Poorly	21 (38.9 %)	23 (46.9 %)
Disease status		
Recurrent	4 (7.4 %)	18 (36.7 %)
Primarily metastatic	50 (92.6 %)	31 (63.3 %)
Liver only metastasis		
Yes	10 (18.5 %)	8 (16.3 %)
No	44 (81.5 %)	41 (83.7 %)
Number of metastatic sites		
1	43 (79.6 %)	41 (83.7 %)
2 ≤	11 (20.4 %)	8 (16.3)
KRAS status		
Mutant	3 (5.6 %)	5 (10.2 %)
Wild	51 (94.4 %)	44 (89.8 %)

### ETS and tumor response

A total of 53 patients (51.4 %) showed ETS 6 weeks after treatment, 22 (40.7 %) in the GEMOX group and 31 (63.2 %) in the GEMOX plus erlotinib group (*p* = 0.03) (Table 2). Of the 54 patients who received GEMOX alone, 3 patients had achieved objective response (5.6 %) at the first response evaluation 6 weeks after treatment and 10 patients had achieved overall response (18.6 %). The patients receiving GEMOX plus erlotinib showed a significantly better objective response rate (14/49, 30.6 %, *p* < 0.01) and overall response rate (20/49, 40.8 %, *p* = 0.02) during the same 6-week follow up. Additionally, ETS was significantly correlated with overall response (correlation coefficient, 0.529; *p* < 0.01) (Table 3).

**Table 2:** Overall response rate and early tumor shrinkage 6 weeks after treatment

	All (*n* = 103)	GEMOX (*n* = 54)	GEMOX plus erlotinib (*n* = 49)	*p*-value
Early tumor shrinkage at 6 weeks				
10 %≤	53 (51.4 %)	22 40.7 %)	31 (63.2 %)	0.03
Response at 6 weeks (RECIST)	18 (17.4 %)	3 (5.6 %)	15 (30.6 %)	0.00
Complete response	0 (0.0 %)	0 (0.0 %)	0 (0.0 %)	
Partial response	18 (17.4 %)	3 (5.6 %)	15 (30.6 %)	
Stable disease	72 (69.9 %)	44 (81.5 %)	28 (57.1)	
Progressive disease	13 (12.6 %)	7 (13.0 %)	6 (12.2 %)	
Overall response (RECIST)	30 (29.0 %)	10 (18.6 %)	20 (40.8 %)	0.02
Complete response	1 (0.9 %)	1 (1.9 %)	0 (0.0 %)	
Partial response	29 (28.1 %)	9 (16.7 %)	20 (40.8 %)	
Stable disease	50 (48.5 %)	32 (59.3 %)	18 (36.7 %)	
Progressive disease	23 (22.3 %)	12 (22.2 %)	11 (22.4 %)

**Table 3:** Correlation between early tumor shrinkage 6 weeks after treatment and overall response

		Overall response		Sum
		Response	Non-response	
Early tumor shrinkage	Yes	17	6	23
	No	13	67	80
Sum		30	73	103

(Correlation coefficient: 0.529, *p* < 0.001)

### PFS and OS according to ETS

**Fig. 2:**
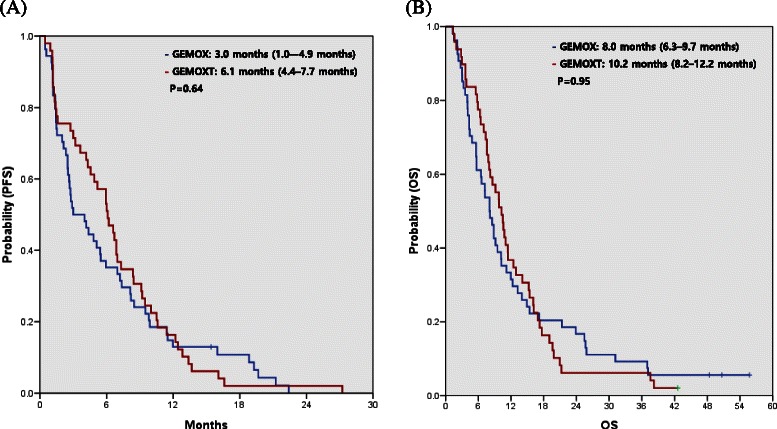
Progression-free survival (PFS) (**a**) and overall survival (OS) (**b**) of patients treated with gemcitabine and oxaliplatin (GEMOX) alone or GEMOX with erlotinib (GEMOXT)

**Fig. 3:**
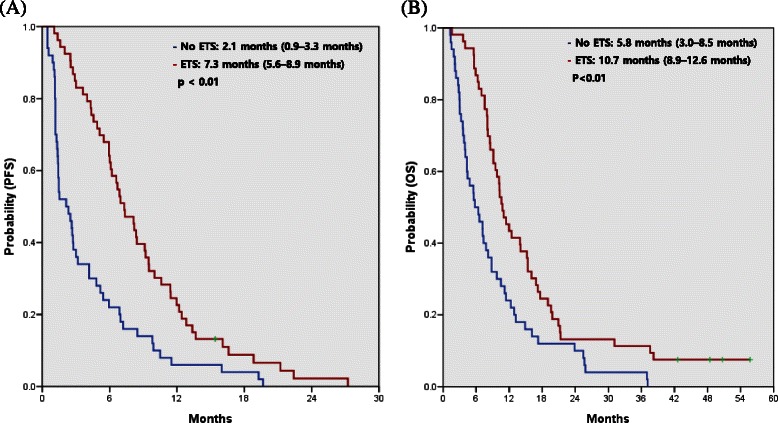
Progression-free survival (PFS) (**a**) and overall survival (OS) (**b**) for patients with or without early tumor shrinkage (ETS) 6 weeks after treatment

There was no statistically significant difference in either PFS or OS (log-rank test, *p* = 0.64 and 0.95, respectively) between the GEMOX alone and GEMOX with erlotinib groups (Fig. 2). In the GEMOX group, the median PFS was 2.5 months (95 % confidence interval [CI], 1.7–3.2 months) for patients without ETS and 5.4 months (95 % CI, 2.0–8.9 months) for patients with ETS (*p* = 0.03, Table 4). There was also a significant difference in OS between patients with and without ETS (9.5 months vs. 4.8 months, *p* = 0.03). In the GEMOX plus erlotinib group, the median PFS was 1.3 months (95 % CI, 1.0–1.6 months) for patients without ETS and 8.3 months (95 % CI, 5.7–11.0) for patients with ETS (*p* < 0.01, Table 4). OS was also significantly different between patients with and without ETS (11.4 months vs. 6.4 months, *p* < 0.01). The median PFS (7.3 vs. 2.1 months, *p* < 0.01) and OS (10.7 vs. 5.8 months, *p* < 0.01) were significantly longer amongst patients with ETS at 6 weeks, irrespective of the treatment received (Fig. 3).

**Table 4:** Kaplan-Meier median progression-free survival (PFS) and overall survival (OS) estimates for patients with and without early tumor shrinkage

		Early tumor shrinkage at 6 weeks		*p*-value
		<10 %	≥10 %	
GEMOX	PFS (months) median (95 % CI)	2.5 (1.7-3.2)	5.4 (2.0-8.9)	0.03
GEMOX plus erlotinib	PFS (months) median (95 % CI)	1.3 (1.0-1.6)	8.3 (5.7-11.0)	0.00
Overall	PFS (months) median (95 % CI)	2.1 (0.9-3.3)	7.3 (5.6-8.9)	0.00
		Early tumor shrinkage at 6 weeks		*p*-value
		<10 %	≥10 %	
GEMOX	OS (months) median (95 % CI)	4.8 (1.6-7.9)	9.5 (7.5-11.4)	0.03
GEMOX plus erlotinib	OS (months) median (95 % CI)	6.4 (3.1-9.6)	11.4 (7.6-15.2)	0.00
Overall	OS (months) median (95 % CI)	5.8 (3.0-8.5)	10.7 (8.9-12.6)	0.00

### Impact of ETS and erlotinib treatment in patients with wild-type *KRAS* tumors

**Fig. 4:**
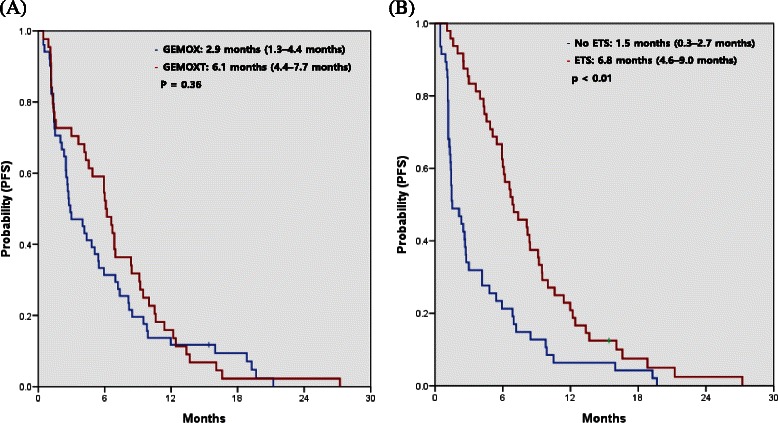
Progression-free survival (PFS) according to treatment with gemcitabine and oxaliplatin (GEMOX) or GEMOX with erlotinib (GEMOXT) (**a**) and early tumor shrinkage (ETS) 6 weeks after treatment (**b**) in patients with wild-type *KRAS* tumors

**Fig. 5:**
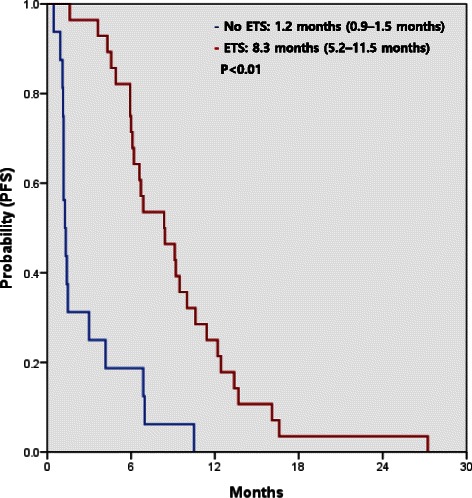
Progression-free survival (PFS) in patients with wild-type *KRAS* tumors treated with gemcitabine and oxaliplatin plus erlotinib (GEMOXT), stratified according to early tumor shrinkage (ETS)

In a subgroup analysis of 95 patients with wild-type *KRAS* tumors, the median PFS was not significantly different between treatment groups (2.9 months for the GEMOX group vs. 6.1 months for the GEMOX plus erlotinib group, *p* = 0.36), but this was significantly longer in patients with ETS than in those without ETS (6.8 months for ETS vs. 1.5 months for no ETS, *p* < 0.01) (Fig. 4). ETS was more strongly associated with PFS in patients with wild-type *KRAS* tumors who were treated with erlotinib (8.3 months for ETS vs. 1.2 months for no ETS, *p* < 0.01) (Fig. 5).

## Discussion

This analysis of the previously reported GEMOX and erlotinib trial for BTC demonstrated that ETS 6 weeks after first-line GEMOX treatment either with or without erlotinib correlates with PFS and OS. The median PFS and OS for patients with ETS were significantly longer than those for patients without ETS, irrespective of the treatment regimen (7.3 vs. 2.1 months, *p* < 0.01, and 10.7 vs. 5.8 months, *p* < 0.01, respectively). Although a strong association between ETS and long-term outcome has been reported in patients with metastatic CRC [8, 11, 12, 14], this is the first study to demonstrate such a relationship in BTC patients.

Selecting patients who will benefit from anti-cancer therapy is an important goal. Biomarkers of response and long-term survival benefit from palliative chemotherapy are urgently needed for various cancer types for the rational and effective use of drugs. In our phase III trial (NCT01149122) of GEMOX with or without erlotinib, performance status, primary tumor site, and metastasis limited to the liver were assessed as potential prognostic factors for long PFS irrespective of the treatment regimen [6]. However, these factors are already included in the patients’ baseline characteristics and cannot help guide treatment decisions, including whether to continue or discontinue therapy. ETS is a known prognostic parameter for the outcome of metastatic CRC patients with wild-type *KRAS* tumors receiving cetuximab [8, 11], and a number of studies have demonstrated a relationship between ETS and clinical outcomes after cetuximab therapy for pretreated metastatic CRC [8, 11, 13–15]. Thus, early changes in response to treatment could help identify patients who would benefit from a continuation of therapy. To date, there have been no effective surrogate biomarkers for predicting response and survival outcome after treatment in advanced BTC patients. Our findings suggest that ETS at 6 weeks might to be a good predictive marker for long-term outcomes in advanced BTC and could guide on-treatment decisions including continuation or discontinuation of therapy, although further confirmation by a prospective trial is needed.

We used a cutoff value of a 10 % decrease in tumor size at 6 weeks as the criterion for ETS. This value was previously used as a cutoff to predict improved outcome in Choi’s criteria for gastrointestinal stromal tumors treated with imatinib and metastatic CRC treated with cetuximab [8, 12, 16]. The significance of this apparently rather small decrease might be related to the number of cancer cells actually eradicated by treatment; in a spherical tumor, 10 % shrinkage would indicate that almost 30 % of cells have been killed [17, 18].

There is growing evidence that the EGFR pathway is a potential therapeutic target in BTC [19, 20]. Although *KRAS* mutations are associated with less efficient EGFR-directed targeted therapy in various cancer types, it is not yet known if the same is true in BTC [21, 22]. Previously, we assessed whether the *KRAS* status could act as a predictive biomarker in patients with advanced BTC who received erlotinib, and this suggested that the *KRAS* mutation might be a predictor of resistance to small-molecule EGFR inhibitors. In present analysis, GEMOX plus erlotinib group included only 5 patients with KRAS mutant tumor. Thus, we could not evaluate the role of KRAS status as a biomarker to erlotinb. Instead, we found a strong association between ETS and long-term outcome in patients with wild-type *KRAS* BTC tumors who were treated with first-line chemotherapy plus erlotinib. This suggests that ETS might help identify a distinct subgroup of advanced BTC patients with wild-type *KRAS* tumors who could benefit from erlotinib treatment.

This study had several limitations. First, this analysis was available in only 103 out of 268 patients who had been enrolled in our phase III trial. Moreover, the subgroups were relatively too small. Small sample size and selection bias of the current study may make definitive conclusions difficult. Second, we retrospectively evaluated only one time point (the follow-up at 6 weeks), which was defined in the study protocol. We are not sure if this time point is optimal for measuring early tumor changes. Therefore, validation in a prospective trial with ETS measured at various time points is needed. Third, because it is well known that extensive desmoplasia and surrounding inflammation in BTC make it difficult to measure tumor responses accurately using conventional methods, new technology for evaluating tumor bioactivity such as PET-CT may allow the treatment effect to be measured more precisely.

The rarity of BTC hinders clinicians from conducting definitive trials and from producing rigorous scientific data. Thus, coordination of trials among institutions and cooperative groups, both nationally and internationally, will be the key to improving treatment outcomes in BTCs.

## Conclusion

ETS 6 weeks after treatment is a possible predictive marker of PFS and OS in advanced BTC. Further to our analysis, we also propose that ETS may help determine whether the addition of erlotinib to chemotherapy would be beneficial to BTC patients with wild-type *KRAS* tumors. These findings need to be prospectively validated.
